# Overview of host miRNA properties and their association with epigenetics, long non-coding RNAs, and Xeno-infectious factors

**DOI:** 10.1186/s13578-021-00552-1

**Published:** 2021-02-25

**Authors:** Samaneh Heydarzadeh, Maryam Ranjbar, Farokh Karimi, Farhad Seif, Mohammad Reza Alivand

**Affiliations:** 1 Department of Medical Genetics, Faculty of Medicine, Tabriz University of Medical Sciences, Tabriz, Iran; 2 Immunology Research Center, Tabriz University of Medical Sciences, Tabriz, Iran; 3 Department of Biotechnology, Faculty of Science, University of Maragheh, Maragheh, Iran; 4 Department of Immunology and Allergy, Academic Center for Education, Culture, and Research (ACECR), Tehran, Iran; 5 Neuroscience Research Center, Iran University of Medical Sciences, Tehran, Iran

**Keywords:** MicroRNA, IsomiR, Arm selection, DNA methylation, LncRNA, Xeno-infection

## Abstract

MicroRNA-derived structures play impressive roles in various biological processes. So dysregulation of miRNAs can lead to different human diseases. Recent studies have extended our comprehension of the control of miRNA function and features. Here, we overview some remarkable miRNA properties that have potential implications for the miRNA functions, including different variants of a miRNA called isomiRs, miRNA arm selection/arm switching, and the effect of these factors on miRNA target selection. Besides, we review some aspects of miRNA interactions such as the interaction between epigenetics and miRNA (different miRNAs and their related processing enzymes are epigenetically regulated by multiple DNA methylation enzymes. moreover, DNA methylation could be controlled by diverse mechanisms related to miRNAs), direct and indirect crosstalk between miRNA and lnc (Long Non-Coding) RNAs as a further approach to conduct intercellular regulation called “competing endogenous RNA” (ceRNA) that is involved in the pathogenesis of different diseases, and the interaction of miRNA activities and some Xeno-infectious (virus/bacteria/parasite) factors, which result in modulation of the pathogenesis of infections. This review provides some related studies to a better understanding of miRNA involvement mechanisms and overcoming the complexity of related diseases that may be applicable and useful to prognostic, diagnostic, therapeutic purposes and personalized medicine in the future.

## Introduction

MicroRNAs (miRNAs) with 18–25 nucleotides are highly conserved non-coding (NC) RNAs that can be found in *C. elegans* to *homo sapience* and can play vital roles in the regulation of gene expression [1, 2]. The locations of human miRNAs are almost in intergenic and intragenic or intronic regions of the genome [3]. The maturation of miRNAs is done by Drosha and Dicer as RNase III enzyme, from the nucleus to the cytoplasm of cells [4]. Then, these miRNAs are associated with Argonaute (Ago) protein to produce the effector RNA-induced silencing complex (RISC) and contribute to the RISC complex to scan the related targets [5]. The unique and important role of miRNAs refers to post-transcriptional regulation by endonucleotide cleavage or inhibition of mRNA translation through the formation of miRNA- induced silencing complex (miRISC) on target sites in the 3′ untranslated region (UTR) of mRNAs [6]. Each miRNA can target multi mRNAs. Moreover, each transcript can be targeted by various miRNAs simultaneously. Although the role of miRNAs in ongoing biological processes, including apoptosis, metabolism, differentiation [7, 8], signal transduction [9], and other normal function of the cell has been demonstrated by numerous studies, their dysregulation leads to disruption of mRNA expression profiling in various disease processes [10, 11], organ transplant rejection [12], rheumatoid arthritis [13], cardiovascular diseases [14], diabetes [15], etc., particularly cancer development [16] and viral infections [17]. It makes miRNAs potential targets for cancer therapies, prognostic biomarkers, or diagnostic signs in diverse diseases.

MiRNAs have several distinct features compared to other functional RNA species. In this regard, diverse variants of a miRNA's so-called isomiRs can affect the miRNA target selection. They are complex property of miRNAs that play functional roles in some diseases such as lipid homeostasis [18], Alzheimer's disease [19], and different cancers [20]. Also, the next-generation sequencing (NGS) studies have shown that isomiRs circulate in the bloodstream with high stability similar to mature miRNA. Thus they might act as novel biomarkers along with miRNAs for malignancies [21]. Arm selection and arm switching are the other remarkable properties of miRNAs. which impact on miRNA function and their imbalance has been considered by many scientists as a significant issue in finding the cause of diseases such as cancer [22, 23]. It is important that although two arms of pre-miRNA (5p and 3p) are relatively complementary [24], each arm includes various related isomiRs that the functional arm involves target detection based on their affinity to RISC complex [25].

Regulation of miRNA expression through molecular epigenetic mechanisms is another important subject in the field of miRNAs and pathogenesis. DNA methylation of miRNA locus and miRNA processing genes leads to the regulation of their expression. On the other hand, multiple miRNAs can target and control the methylation-related enzymes and factors to affect epigenetics event. Aberrant expression of miRNA mediated by epigenetics and aberrant activity of DNA methylation enzymes mediated by miRNA are important in the pathogeneses of diseases [26]. It can provide a strategy for early diagnosis and treatment of cancer in vitro and in vivo [27]. Additionally, the interaction between miRNA and lnc-RNAs as competing endogenous RNA control miRNA-mRNA binding and have therapeutic importance in cancer [28].

Valuable studies confirmed that there is a significant correlation between miRNAs and some infectious factors and agents; therefore, it can affect the progress or regress of these diseases [29–31]. There are applicable issues mentioned above to plan future direction in controlling diseases. In this review, we first provide a brief overview of some miRNA properties from variation in their sequence (isomiR) to regulation of miRNA arm selection/switching and the effect of some factors on these two features. We then collected some evidence about the relationship between miRNA and epigenetics, long non-coding RNAs, and Xeno-infectious factors. Altogether, the regulation of miRNA function mediated by these factors is an important issue; therefore, it can provide new insight into the etiology of diseases and their treatment.

### miRNA properties

The miRNA sequences and their maturation had been conserved from primary to higher eukaryotes. The primary transcript of miRNAs (≤ 100 bp) is processed using a two-step mechanism with two RNase III enzymes in the nucleus and cytoplasm. Firstly, Drosha in collaboration with DiGeorge syndrome critical region 8 (DGCR8) accessory protein as a microprocessor complex binds to double-stranded miRNA and cleaves it to the generate pre-miRNA (~70 bp) [32]. After transmission of pre-miRNA to the cytoplasm using the Exportin-5 and Ran-GTP complex, the subsequential processing of miRNA is done by Dicer as the second RNase III and generates the matured double-strand miRNA (20–23 bp). RISC-loading complex includes double-stranded RNA, Dicer, the trans-activating response RNA-binding protein (TRBP), and Argonaut 2 that is essential for activation of RISC complex to follow related mRNAs using suitable single-stranded miRNA as guide RNA [33]. The discovery of the frequency of miRNAs in various multicellular species raised intriguing questions, including what these molecules may do in the cell. The key response is to find their mRNA targets. In other words, highly conserved miRNAs have extremely conserved targets. In the miRNA, there are several interaction sites with target mRNAs to direct post-transcriptional repression. Many sites that match the miRNA seed region (nucleotides 2–7), particularly those in 3′UTRs, are preferentially conserved. Four types of these sites are 6mer site, which perfectly matches the 6-nt miRNA seed, 7mer-m8 site that involves the seed match complete by a Watson–Crick match to miRNA nucleotide 8, 7mer-A1 site, which contains the seed match supplemented by an A across from miRNA nucleotide 1, and 8mer site, which includes the seed match supplemented by both m8 and the A [34]. Furthermore, experiments using artificial sites show that targeting can also occur in 5′ UTRs, especially in open reading frames (ORFs) [35].

In silico study of miRNAs, like the other members of the genome, requires databases. Recently, five criteria databases have been developed. At first, miRBase was developed, which provided nomenclature for newly discovered miRNA genes, thereby making available the annotation and sequences of all published miRNAs from various organisms to researchers [36]. MirGeneDB 2.0 is a manually created metazoan miRNA gene database that has a more complementary nomenclature system than MiRBase. Also, it contains previously overlooked miRNAs and seven organisms that are not currently listed in miRBase. However, it contains fewer entries than miRBase [37]. MiRCarta is another miRNA database, which provides collection of validated novel human miRNAs and enhances the information provided by miRBase. In fact, miRCarta illustrate a more inclusive companion to manually created resources such as MirGeneDB [38]. Also, miRTarBase (http://miRTarBase.mbc.nctu.edu.tw/), miRDB (http://mirdb.org), miR-EdiTar (http://microrna.osumc.edu/mireditar), TransmiR v2.0 (http://www.cuilab.cn/transmir), miRandb (http://mirandb.ir), HMDD v3.0 (http://www.cuilab.cn/hmdd), and ImmunemiR (http://www.biominingbu.org/immunemir/) are the other important databases in the field of miRNA.

### miRNA isomiRs

**Fig. 1:**
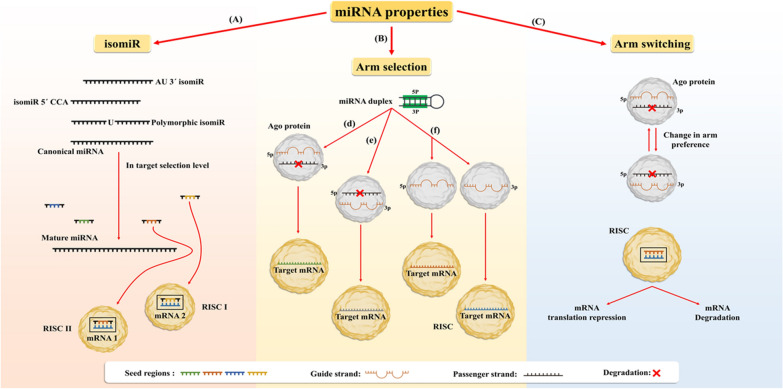
MiRNA properties. **a** IsomiRs as miRNA variants are categorized into three main types, including 3′ isomiR, 5′ isomiR, and polymorphic isomiR that they can affect a miRNA at the target level to select different mRNA (**b**) Arm selection is a feature of miRNA that can affect target selection of miRNA by isomiRs and it can occur in three common steps, including (**d**) 5p arms of a miRNA duplex selected as a guide strand to incorporates into the RISC complex., (**e**) 3p arms of a miRNA duplex may be selected as a guide strand to incorporates into the RISC complex., and (**f**) occasionally, two arms of a miRNA duplex can be used as guide RNA and incorporate into the RISC complexes with different target mRNAs. Note: the guide strand of a miRNA has weaker binding to Ago protein. However, the passenger strand has a tighter binding capacity to Ago protein. **c** Arm selection of a miRNA can be changed in different conditions that it termed as arm switching

Mature miRNAs consist of various variants that are diverse in length and/or sequence so-called isomiRs [39]. For the first time, isomiRs of miRNAs were determined by RNA-Seq approaches and it has been observed that any miRNA has different isomiRs with multiple copy numbers. IsomiRs are classified into three main types, including 3′, 5′ and polymorphic isomiRs with different nucleotide sequences compared with canonical miRNA (Fig. 1) in which 3′ isomiRs are most common in animals and plants [40–42] (Fig. 1a). Both 5′ and 3′ isomiRs are divided into homogenous (template) and or heterogeneous (non-template) variants. Polymorphic isomiRs are known as non-template sequences. The difference between these variants is attributed to their sequence that is match or non-match with the genome (parent gene) [41].

There are some mechanisms to generate these various isomiRs. Processing heterogeneity as the main process is involved in the generation of 5′ and or 3′ template isomiRs, provided by Drosha and or Dicer imprecise cleavage in the 5′ and or 3′ ends. A further mechanism is nucleotide trimming mediated by some exoribonucleases or some nucleotidyltransferases that are less common for the generation of template isomiRs in animals, bacteria, humans, and others. The mentioned mechanisms are common in 3′ end rather than 5′. Additionally, post-transcriptional enzymatic processes that made non-template isomiRs are comprised of (1) nucleotide addition by ribonucleotidyl transferase mainly uridyltransferase and adenyltransferase [43, 44], (2) nucleotide removal, which these two changes often occur in 3′ isomiRs, (3) adenosine (A) to inosine (I) RNA editing by the double-stranded RNA-specific adenosine deaminase (ADR) enzyme that is the most prevalent RNA-editing enzyme in isomiRs. RNA editing may occur in the seed region of 5′ end, in the 3′ end nucleotides or internal nucleotides and generate 5′, 3′ and polymorphic isomiRs, respectively. This mechanism is the major process in the generation of polymorphic isomiRs [41, 45]. According to different pieces of evidence, all of the generated modifications could affect different functions of a miRNA such as a target selection, efficiency to target in RISC complex, diversity, stability, and half-life of miRNA [46–51].

Based on isomiR profiling, the isomiR variation of a miRNA is related to cells and tissue types [52]. Also, they are associated with the individual’s gender, population origin, and race that all of them dependent on a dynamic and regulated maturation process that originated from the response to various biological inductions [53, 54]. More recent studies demonstrated that specially derived isomiRs could be applied as more effective prognostic and diagnostic biomarkers in various cancers and related subtypes, thus they can differentiate healthy and non-healthy individuals. For example, isomiR-140-3p is confirmed in the prevention of triple-negative subtype of breast cancer and there are ongoing studies regarding the prediction of further disease [55]. Also, different isomiRs of miR-222 could play distinct roles, as a result, it may be used as a biomarker in breast cancer cells and other tumors [57]. These observations further support the fact that diverse isomiRs from the same mature miRNA can target practically non-overlapping collection of transcripts. Accordingly, so far, some reliable approaches have developed with high sensitivity to the detection of isomiRs that could facilitate this detection. One of the novel applications is Quagmire with a flexible searching algorithm that analyzes heterogeneous isomiRs from next-generation sequencing data and identifies different isomiRs [58]. Recently, Comprehensive Approach to Sequence-oriented isomiR annotation (CASMIR) database that facilitates precise interpretation of isomiR features in small RNA sequencing data among species and miRDeep2, which is a favorite algorithm of miRNA analysis to accredit isomiR interpretation by CASMIR, are two ways of analysis of isomiRs [59].

Several tools have been developed to analyze miRNAs and their respective isomiRs that these are different in their abundance cut-offs, isomiR annotation methods, ways to handle cross-mapping events, or alignment strategies. A number of bioinformatics tools only can analyze and detect isomiRs without their functional annotation, including CPSS, IsomiRex, MODOMICS, MiRGator v3.0, and SeqBuster. In the meantime, SeqBuster is a reliable, more flexible and highly versatile web-based toolkit due to overcoming storage capacity limitations through providing a stand-alone version that can permit the annotation against any custom database [60–64]. Up to now, SeqBuster, miRspring, isomiRex, YM500v2, YM500, RNASEQR, and miRGator v3.0 as isomiR databases have been used to diagnose cancer [65]. IsomiR-SEA as a novel tool based on RNA-Seq analysis can accurately detect miRNAs/isomiRs expression level profiling and evaluate conserved miRNA-mRNA interaction sites [66]. SRNAbench is the other high-throughput analytic tool for profiling of miRNAsʼ isomiRs in one or simultaneously multiple species [67]. MiRge 2.0 is a tool, which widely analyzes miRNA sequencing data and exclusively captures the potential miRNAs by using both composition of isomiRs and miRNA hairpin sequence structure [68]. The software PRocessing Of Short Transcripts (Prost), helps quantify mature miRNAs that accounts for post-transcriptional processing such as nucleotide editing and identifies mirror-miRNAs [69]. Additionally, there are some other platforms or software to study miRNA/isomiR such as miR-isomiRExp, miRWalk2.0, DIANA miRPath v.2.0, mirBridge, GeneSet2miRNA, miRror2.0, and C2Analyzer [70].

Recently, an optimized miRNA analysis project called miRNA Transcriptomic Open Project (miRTOP) has been designed. The purpose of miRTOP is to develop the downstream isomiR analysis tools that are compatible with available quantification and detection tools. Also, it solves the lack of consensus between related tools and allows any tool to convert results into mirGFF3 format as a standardized output format for miRNAs/isomiR analyzing [71].

### Arm selection and arm switching of miRNAs

Before scanning and selecting the target by miRNA, there are two steps that add complexity and specificity to its roles. One of them is “arm selection” as a highly controlled process. In this regard, each miRNA is formed from 3 and 5p arms of precursor miRNA (pre-miRNA). Although the derived 3p and 5p arms of miRNA are mostly complementary and derived from the same transcript, they result in different isomiR expression profiles and patterns under different situations; for example, each 5p and 3p arms could participate in various RISC complexes as guide strands or one of the involved arms is degraded as a passenger RNA in the RISC complex (Fig. 1b) [25, 72–74]. Therefore, some factors can be involved in guide strand selection of RISC, including lower thermodynamic stability at the 5′end and its weaker binding of 5′ end of a strand to the AGO2 protein to direct specific RISC toward the target gene. It seems AGO2 protein plays a major role in this process [75, 76]. Additionally, any post-transcriptional modifications on the 5′- or 3′-end of each strand of miR duplex can considerably affect arm selection [41, 77–79]. Also, the frequency of isomiRs can be important in involving the selected arm. For example, a study showed that affluence of miR-140-3p isomiRs, compared with miR-140-5p, leads to the formation of a novel seed region in 3p arm, which is more functional than the original consensus one to detect targets in human cartilage cells [80].

Furthermore, some other factors can determine guide RNA selection, such as U/C base at the 5′-end of a strand and an excess of purines/pyrimidines in guide RNA [81] that are directly or indirectly derived from different expression levels of protein activator of dsRNA-dependent protein kinase (PACT), DICER, TRBP and 5′-3′ exoribonuclease (Xrn)-1/2 in various cells and tissue types [82, 83] as well as the abundance of target's copy numbers may contribute to arm preference [84]. Another aspect of miRNA is related to miRNA’s biological ability to switch strand preference called “arm switching” (Fig. 1c). The loaded arms of RISC are related to the frequency of them. Arm switching events are different at various differentiation states, various cells and tissue types (such as a variety of species and gender) possibly due to alternative Dicer cleavage or Drosha processing [85–87]. Thus, arm switching is a mechanism that plays vital roles in the evolution of miRNA gene functions under different conditions in mRNA targeting [25, 88, 89].

Accordingly, some reports showed that both arms of a miRNA can conduct distinct targets and play different roles in cancer. For instance, both miR-193a-3p and miR-193a-5p expression decrease in gastric cancer cells and their ectopic expression show that miR-193a-5p inhibited these cells’ growth, but only miR-193a-3p remarkably repressed cell invasion via directly targeting ETS1 and CCND1 expression [90]. Furthermore, miR-324-3p and -5p were significantly overexpressed in lung cancer cells, so their ectopic expressions have different effects on lung cancer cell line and the overexpression of miR-324-3p only enhance cell proliferation but did not alter the invasion of these cells, while miR-324-5p significantly promoted both cell invasion and proliferation[92]. Altogether, the arm selection and/or arm switching have key functions in the regulation of isomiRome and miRNAome profiles and lead to changes in isomiR/miRNA expression profiles to evolutionary and/or functional pressures. Also, these results suggest that miRNAs arm switching and or arm selection could be the other essential mechanism of miRNA variation and applicable biomarkers in various diseases such as cancers.

### Crosslinking between DNA methylation and miRNAs

DNAmethylation is a dynamic and reversible event that its status depends on the regulation of such involved enzymes through further factors. Accordingly, such documents demonstrated that miRNAs could target and control the mentioned enzymes. Moreover, various miRNAs located in CpG islands and shores could be controlled epigenetically. Although DNA methylation could impact the expression of miRNAs via their promoter region, it can regulate the expression of miRNA processing-related enzymes [57, 92–94]. Therefore, DNA methylation affects both miRNAs and processing related enzymes. Some miRNAs such as miR-29 family (29a, 29b, and 29c) could target directly and indirectly DNA methylation-related enzymes such as TETs and DNMTs, and histone-modifying enzymes; for example, histone deacetylase 4 (HDAC4) and histone methyltransferase SET domain bifurcated 1 (SETDB1) are impaired in various cancer e.g., lung cancer, breast cancer, hepatocellular cancer, etc. [95–102]. Additionally, a study indicated that as miR-29 could control the other miRNAs expression such as miR-34c and miR-449a by targeting DNMT3a and 3b [103]. Other studies established that miR-29b modulated the global DNA methylation through targeting DNMT3A and DNMT1, leading to decreased transcription factor specificity protein 1 (SP1) and increased p21 expression in chronic lymphocytic leukemia (CLL) cells [104]. MeCP2 is one of the other target genes for miRNA-29a as an epigenetic mediator. Overexpression of MiR-29a leads to a decrease in the Bromo domain-containing protein 4 (BRD4) signaling and zinc finger protein SNAI1 expression and downregulated methyl-CpG-binding protein 2 (MeCP2) in mouse hepatic stellate cells (HSCs) [105]. It seems the miR-29 family plays a vital role in the modulation of epigenetic phenomenon compared with other associated miRNAs.

One research declared that co-transfection and overexpression of miR-339 and miR-766 lead to inhibition of DNMT3b upregulation in colon cancer. Subsequently, it results in reactivating the expression of such tumor suppressor genes SFRP1, SFRP2, DKK2 and WIF1 in these cells [106]. MiR-221 is another miRNA involved in DNMT3b targeting, which can elevate the cancer stem cell properties such as *Oct3/4* and *Nanog* through downregulation of DNMT3b in breast cancer cell lines [107]. According to a study, both mir-148b and mir-152 can reactivate some tumor suppressor genes such as SPARC and BNIP3 by targeting DNMT-1, thereby resulting in modification of methylation status of the mentioned tumor suppressor genes and reducing tumorigenic properties in pancreatic cancer cell lines [108].

To the best of our knowledge, there are two studies on miRNA-140 and methylation regulation. Accordingly, miR-140, as a tumor suppressor, controls NF-κB activity by direct targeting Dnmt1 and conducting hypomethylation and overexpression of metallothionein genes to indirectly enhance NF-κB activity in a liver cell line [109]. The second one shows that miR-140-5p can regulate CD4^+^ T cell differentiation through demethylation of GATA3 and hypermethylation of STAT1. In addition, it is involved in the tricarboxylic acid (TCA) cycle by the regulation of methylation status of mediated transcription factors that may be associated with TET2 activities. Therefore, mir-140 probably performs its epigenetic roles by controlling both Dnmt1 and TET2 [110]. Based on in vitro and in vivo experiments, there are downregulated and upregulated miRNAs such as miR-212, miR-373, miR-638, miR-106a, miR-221/222, miR-19a/b, miR-132, miR-7b, miR-130a, miR-22, miR-483-5p and miR-218 that may control the expression level of Methyl-CpG-binding protein MECP2 as a reader of DNA methylation [111–115,117–124]. Furthermore, Methyl-CpG-binding domains (MBDs) i.e., MBD1 and MBD2 could be regulated through miR-195-5p, miR-224 in various diseases. Therefore, DNA methylation could be modulated by miRNAs involved in targeting of MBD proteins [125, 126] (Table 1).

**Table 1:** Effect of the related miRNAs on DNA methylation

miRNA	miRNA expression	Outcome	Disease	Refs.
miR-29 family	Down-expressed	Decrease targeting DNMT1, 3A, 3B and TET1	Multiple myeloma, lung cancer, Burkitt lymphoma, breast cancer, nasopharyngeal carcinoma	[79–81, 95, 97–100, 102]
miR-339 and miR-766	Down-expressed	Decrease targeting DNMT3B gene	Colorectal cancer	[11, 108, 107, 113–115, 122, 124, 183]
miR-221	Over-expressed	Increase directly targeting DNMT3b 3′UTR region	Breast cancer	[184]
miR-148b and miR-152	Down-expressed	decrease targeting DNMT-1 mRNA	Pancreatic cancer	[185]
miR-140	Down-expressed	Decrease directly targeting DNMT-1	Liver cancer	[90]
miR-140-5p		Decrease DNA methylation of STAT1 and Tbx genes CpG island	Autoimmune encephalomyelitis	[186]
miR-212	Down-expressed Down-expressed	Increase MeCP2 protein level	Gastric cancer	[187]
miR-373	Down-expressed	Increase MBD2 expression	Hilar cholangiocarcinoma	[188]
miR-638	Down-expressed	Increase MeCP2 mRNA level	Gastric cancer	[189]
miR-221/222	Down-expressed	Increase MBD2 expression	Cervical cancer	[190]
miR-19a/b	Down-expressed	Increase MeCP2 expression	Gastric cancer	[191]
miR-132	Over-expressed	Decrease MeCP2 expression	Chronic Cerebral Hypo perfusion	[192]

According to different studies, some miRNAs are regulated by their methylated locus or promoter region. For example, miR-200c and miR-141 play important roles in the Epithelial-Mesenchymal Transition (EMT) event of solid cancers that both of them are regulated epigenetically [127–131]. In prostate cancer, the promoter region of miR-200c and miR-141 are hypermethylated, leading to downregulation of their expression [132]]. This association between miR-141 and miR-200c is confirmed in gastric and breast cancers, respectively [133, 134]. Another miRNA involved in gastric cancer invasion is miR-7-5p that its silencing by methylation of its promoter leads to an increase in its target genes, namely Smo and Hes1 [135]. Furthermore, the promoter methylation of miR-7 is a significant and early-stage biomarker in cisplatin-resistance and clinical management of ovarian and lung cancer cells [136] (Table 2).

**Table 2:** Effect of DNA methylation on miRNAs

miRNA	Expression	Mechanism	Disease	Refs.
miR-874	Down expressed	Hyper methylation of the promoter region	Breast cancer	[194]
miR-129-2 and miR-9-1	Down expressed	DNA methylation of the miRNA promoter CpG island	Renal cell carcinoma	[75]
miR- 10b-3p	Over expressed	Promoter hypo methylation	Esophageal squamous cell carcinoma	[195]
miR-141	Down expressed	Hyper DNA methylation	Gastric cancer	[108]
miR -145	Over expressed	Demethylation of the promoter region	Breast cancer	[50]
miR-200c and miR-141	Over expressed	Hypomethylation of the promoter region	Colorectal Cancer	[109]
miR-200c/141	Down expressed	Hyper methylation of CpG island located in the promoter region	Invasive breast cancer	[114]
miR-370	Down expressed	Hyper methylation of two CpG islands located in the upstream of miR genomic locus	Osteosarcoma	[196]
miR-941 and miR-1247	Down expressed	Hyper methylation of the CpG island in miRs loci	Gastric cancer	[197]
miR‐7‐5p	Down expressed	Hyper methylation of the promoter site	Gastric cancer	[116]
miR-21 and miR-146b	Over expressed	Hypo methylation of miRs promoter region	Papillary thyroid carcinoma	[198]
miR124-2	Over expressed	Hypo methylation of CpG site in miR gene	Breast Cancer	[199]
*miR-183*	Down expressed	Hyper methylation of the miR promoter	Hepatocellular carcinoma	[200]

**Fig. 2:**
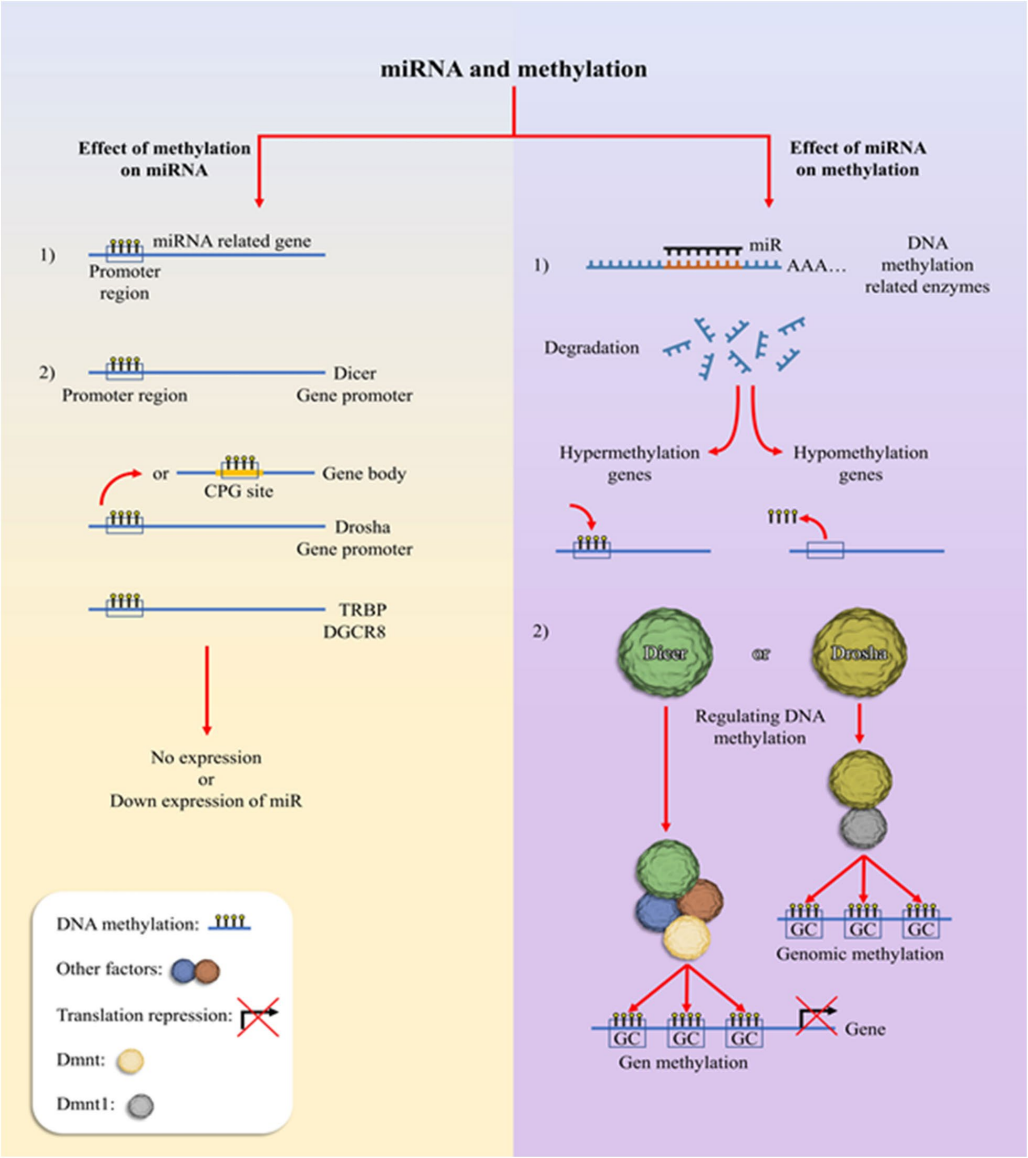
MiRNA and methylation: miRNA and DNA methylation have interaction with each other in two ways, including the effects of miRNA on methylation and vice versa. DNA methylation can affect 1) miRNA-related gene or 2) promoter region of miRNA processing enzymes and cofactors such as TRBP and DGCR8 and also can affect CPG sites of Drosha gene body. Mutually, a miRNA can affect DNA methylation in two ways, including 1) it can target genes of DNA methylation-related enzymes such as DNMTs, TETs, MECP2, and MBDs, and leads to hyper- or hypo-methylation of different genes. 2) Also, miRNA processing-related enzymes can directly or indirectly affect DNA methylation through recruiting DNMTs to CpG sites in the genome or at gene levels

Although miRNAs could be controlled epigenetically and vice versa, the expression of miRNA processing enzymes, including DROSHA, DGCR8, EXPORTIN5, Dicer, and TRBP, can be affected by DNA methylation and its related factors directly or indirectly [137] (Fig. 2). According to studies, methylation of some CpG sites in the gene body of Drosha has a significant correlation with the stimulation of transcriptional elongation in cancer cells [138, 139]. The further study reported that MeCP2 binds to DGCR8 and suppresses the DGCR8/Drosha complex directly in the brain [140]. In contrast, Drosha can also affect and maintain DNA methylation by mediating DNMT1 activity [141]. As a result, there is a bilateral association between Drosha complex and DNA methylation. Exportin-5 (*XPO5*), as a master protein, exports pre-miRNAs from nuclear to the cytoplasm. The related study showed that XPO5 promoter methylation status controls its expression level in breast cancer patients [142].

The association of Dicer, as another processing enzyme in DNA methylation, was demonstrated through some studies. Accordingly, in Cholangiocarcinoma (CCA) was declared that the overexpression and translocation of Dicer to the nucleus and formation complex with heterochromatin protein 1α (HP1α) leads to hypermethylation of SFRP1 promoter and suppression of its transcription through recruiting Dnmts [143]. Another study showed that Dicer indirectly could control methylation of Xist promoter in Embryonic Stem (ES) cells through Dnmt3a regulation [144]. There is a relationship between Dicer and methylation status by controlling of DNMTs in various human cancer cells [145]. It seems that Dicer is indispensable to the maintenance of methylation status throughout the genome.

Despite the association of DNA methylation with the expression of various miRNAs, RNA methylation of pri-miRNAs can modify their expression. RNA methylation is a reversible and dynamic event that is mostly performed in m6A position by specific-related enzymes such as ALKBH5 and METTL3 [146, 147]. RNA methylation of various positions in pri-miRNAs could be impacted by the interaction of processing enzymes such as Dicer [148]. Additionally, the expression of METTL3 could be controlled by DNA methylation. Therefore, RNA methylation is regulated by DNA methylation [149]. Although N6-methyladenosine (m6A) modification is more common, 7-methylguanosine (m7G) as a further RNA modification has been reported in miRNA regulation like hypermethylation of let-7 pri-miRNA by METTL1 [150].

### The interaction between miRNAs and lncs

In addition, the specificity, feature, and function of a miRNA can be affected by the structural variants of the miRNA itself or by epigenetics, which play a significant role in the normal function of the cell or diseases; these can be affected by other RNAs, including lncRNAs.

**Fig. 3:**
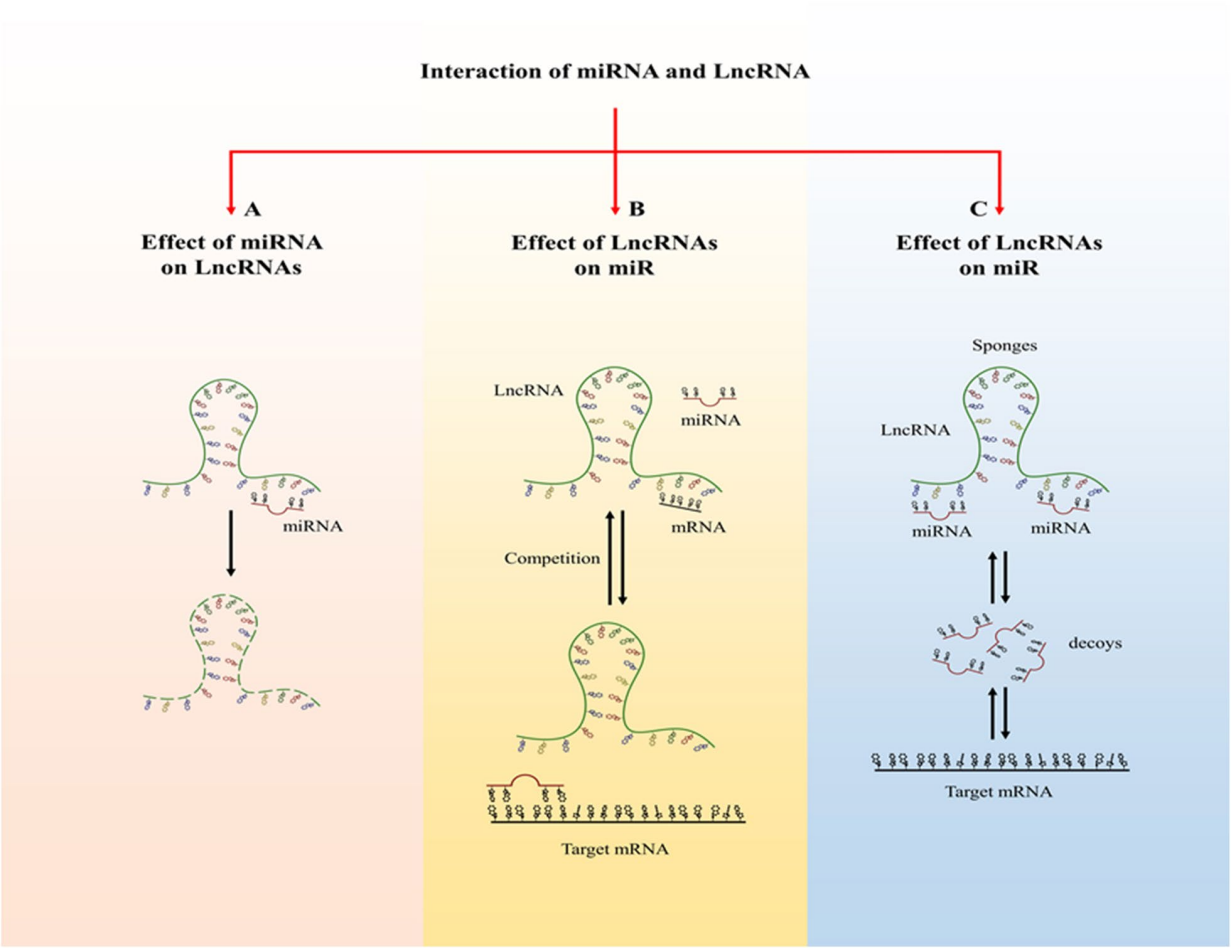
Interaction between miRNA and lncRNA. There are three states of collaboration among miRNAs and lncRNAs: **a** miRNA can bind to its binding site of lncRNA and weak lncRNA stability. **b** LncRNA and miRNA can compete with each other to binding to target mRNA. **c** Also, lncRNA can affect the binding of miRNA to target mRNA through sponging the miRNA. However, sometimes miRNA can decoy from lncRNA and bind to related mRNA

According to significant in vitro and in vivo studies, lnc RNAs as long non-coding RNAs (> 200 bp) involve in many cellular processes and various diseases, which could interact with other non-coding RNAs such as miRNAs to modulate their roles [151]. LncRNAs originate from intragenic and intergenic regions that can activate or repress gene expression at multiple levels through diverse mechanisms, and their interaction with miRNAs is a complex mechanism to regulate target genes. Consequently, the effects of lncRNA-miRNA on the regulation network have attracted extensive attention in medical research [152–154]. This interplay has different aspects. In some cases, miRNAs interact with the miRNA-binding site of lncRNA like their target mRNAs (Fig. 3a), thereby triggering to disturb lncRNAs by miRNAs [155, 156]. In other cases, lncRNAs can compete with miRNAs to bind to the related mRNA (Fig. 3b) or act as miRNA sponges/decoys (Fig. 3c) in some pathway of the cells so-called competing endogenous RNA (CeRNA). Obviously, lncRNA competes with mRNAs for sequestering or binding to miRNAs through matching the miRNA response elements (MREs) [157]. Although there are reports that show the CeRNA abundances alteration from individual genes can modulate the activity of miRNAs, some studies demonstrated the modulation of miRNA target abundance unlikely have significant effects on the gene expression and metabolism through CeRNA [158]. Multiple studies show that lncRNA-miRNA collaboration is the most prevalent collaboration in cancer. In this regard, numerous studies have been performed. For example, lnc-ABCA12-3 is a novel oncogene in esophageal squamous cell carcinoma (ESCC) competes with endogenous miR-200b-3p to regulate the expression of fibronectin 1 (FN1) in metastatic stages of the tumor [159]. Furthermore, the overexpressed lncRNA HAGLROS in hepatocellular carcinoma cell and tissue leads to inhibition of miR-5095 and elevated expression of its target, ATG12 [160]. Another study reported that lncRNA H19 is overexpressed and increases NOX4 expression through miR-148b-3p suppression [161]. This association was observed between different lncRNAs such as MIR205HG, NEAT1, SNHG20, SNHG1, SNHG12, GIHCG, TUSC-7, ATB, MEG3, GAS5, PCF, AL445665.1–4, lnc-p21, LINC00339, TINCR and related miRNAs, including miR-590-3p, miR-129-5p, miR-495, miR-577, miR-16, miRNA-1281, miR-146, miR-141-3p, miR-147, miR-21, miR-344a-5p, miR-146b-5p, miR-625, miR-497-5p, miR-214-5p in normal and cancerous cells, respectively (Table 3) [56, 91, 160, 162–173].

**Table 3:** Interaction between lncRNA and miRNA

LncRNA	miRNA	ceRNA	Disease	Refs.
ABCA12-3	miR-200b-3p	–	Esophageal squamous cell carcinoma	
HAGLROS	miR-5095	Sponging	Hepatocellular carcinoma	[56, 146, 159, 162–168]
MIR205HG	miR-590-3p	Sponging	Head and Neck Squamous Cell Carcinoma	[49]
NEAT1	miR-129-5p	Sponging	Papillary thyroid cancer	[148]
SNHG20	miR-495	Sponging	Breast cancer	[140]
SNHG1	miR-577	Sponging	Osteosarcoma	[142]
SNHG12	miR-16	Sponging	Colorectal cancer	[144]
GIHCG	miRNA- 1281	–	Gastric cancer	[143]
TUSC-7	miR-146	Sponging	Lung adenocarcinoma	[141]
ATB	miR-141-3p	Sponging	Breast cancer	[149]
MEG3	miR-147	–	Chronic myeloid leukemia	[72, 91, 149, 169–173]
GAS5	miR-21	Sponging	Ovarian cancer	[152]
PCF	miR-344a-5p	–	pulmonary fibrosis	[4]
AL445665.1-4	miR-146b-5p	–	Multiple uterine leiomyoma	[153]
lnc-p21	miR-625	–	Neuronal injury	[150]
LINC00339	miR-497-5p	Sponging	Pancreatic cancer	[201]
TINCR	miR-214-5p	Sponging	Hepatocellular carcinoma	[151]

Furthermore, a study demonstrated that lncRNA can inhibit miRNA expression by affecting its promoter region (Fig. 3c); for example, LncRNA-p23154 controls miR-378a-3p expression through this way in oral squamous cell carcinoma [174]. In addition to the studies mentioned above, lncRNA can also affect a miRNA in two ways. For instance, overexpression of LncRNA- miR503HG as a decreasing lncRNA in ovarian cancer (OC) cells may decrease the expression of miR-31-5p via sponging of miR-31-5p and increasing miR-31-5p gene's methylation [175]. Some miRNAs are involved in inhibition of lncRNA activities that may have a reciprocal interaction between lncRNAs and miRNAs; for example, lncRNA XIST and miR-132-3p repress each other in CRC cells [176]. Also, this association was shown in Lnc-OC1/miR-34a/34c of OC and MALAT1/miR-101 of Glioblastoma (GBM) cells, respectively [177, 178]. The mutual interaction between LNC-ZEB1-AS1 and miR-101 was demonstrated in CRC tissues and cells [179]. The growing various in silico and experimental studies help demonstrate the RNA-RNA interactions that functionally impact the related gene regulation derived from detailed primary and secondary structure predictions and their validations. Therefore, synthetic RNAs can have therapeutic applications in the mentioned targets.

### The miRNA and transcript association between xeno-infections and host

Accordingly, another complexity of miRNAs is their effects on xeno-infection’s RNAs that lead to moderate severity and weakness of associated diseases. Moreover, some miRNAs of parasites could probably target the expression of host-related mRNAs because of the conservative sequence of miRNAs from primary to higher organisms. Therefore, the up- and down-regulation of special miRNAs can control the virulence genes in various infections. Also, miRNAs and their related isomiRs can affect or be affected by various populations and immune system activation against infections, especially viral infections that lead to crucial modifications in the miRNA/ isomiR repertoire, which indicates miRNAs’ role and variances of immunity in different hosts alongside the infection [180].

There are several interaction levels between the host’s miRNAs and RNAs of different viruses. Regarding various virus infections, some host’s miRNAs can target critical virus-related mRNAs or part of the virus genome. For example, miR-296-5p is significantly upregulated in enterovirus 71 (EV71)-infected human cells and inhibits replication of the virus by targeting the two regions of the viral genome [181]. Moreover, miR-125b-5p can negatively regulate hepatitis C virus (HCV) infection via targeting Human antigen R (HuR) as an affirmative regulator of HCV replication in both liver carcinoma cells and serum of HCV-infected patients [182]. Also, miR-28-3p inhibits the transmission of human T cell leukemia virus, type 1 (HTLV-1) to T cells by blocking the reverse transcription step of the virus genome [183].

Further studies showed that the number of host miRNAs can influence virus infection by targeting some host factors. For instance, miR-939 decreases the frequency of Hepatitis B virus (HBV) RNAs by targeting host factors like Jmjd3 that it is an enhancer for transcription efficiency of HBV [184]. Also, miR-10a-5p directly targets the signal recognition particle 14 (SRP14) that leads to a decrease in the extracellular viral RNA expression of PRRSV and its multiplication [185]. In a study, gga-miR-29a-3p and gga-miR-19b-3p repressed Newcastle Disease Virus (NDV) multiplication, while the gga-miR-199-5p and gga-miR-451 stimulated this infection.In this regard, it was demonstrated that gga-miR451 performs its role via targeting host factor of tyrosine3 monooxygenase/tryptophan5-monooxygenase activation protein zeta (YWHAZ) [186]. Additionally, hsa-miR-199a has an antiviral effect through the downregulation of a Golgi-localized GTPase-activating protein for Cdc42 called ARHGAP21 in the Herpes Simplex Virus-1(HSV-1) infection [187]. In addition to the above, some miRNAs such as miR-122 play a dual function in the proliferation of Human Papillomavirus (HPV) and Hepatitis C virus (HCV) in infected cell lines [188, 189].

Regarding other studies about the effects of viral factors on the host’s miRNA expression, some virus-related intergenic non-coding RNAsequences of the virus genome can decline the host miRNAs during pathogenesis by affecting the maturation of miRNAs [190]. In a study, the NS3 protein of HCV can upregulate miR-27a; meanwhile, downregulate miR-150 and miR-335 expression in LX-2 liver cells, thereby enhancing the pathogenesis of related diseases [191]. Furthermore, hepatitis B virus X protein (HBx) represses the expression of miR-30e by increasing its promoter methylation that leads to developing hepatocarcinogenesis and liver fibrosis [192]. Also, dengue virus (DENV) and Borna disease virus 1 (BoDV-1) induce hsa-miR-146a overexpression to control the IRAK1/TRAF6/NF-κB signaling pathway in host cells to facilitate viral replication [193]. Other mechanisms of the virus to escape from the host immune system in influenza A virus (IAV) is the mutation of the NS1 gene as a major regulator of pathogenicity that helps to virus proliferation by disrupting the antiviral response of hsa-miR-1307-3p [195]. The appearance of new coronavirus disease 2019 (COVID-19) that has also been termed severe acute respiratory syndrome coronavirus 2 (SARS-CoV-2) with rapid spreading, severe symptoms, which affects the lung, heart, kidney cells, etc. [196–198] by targeting Angiotensin-converting enzyme 2 (ACE2) receptors and the whole world faced a pandemic. Given that the interaction between miRNA and virus materials has been established in various diseases, some miRNAs, which can target capsid protein-coding genes of the virus or ACE2, may be used as a therapeutic solution to inhibit or attenuate COVID-19.

According to the association between miRNAs and microbes involved in the pathogenesis of the disease, there is some gut microbiota such as Proteobacteria, Bacteroidetes, and Firmicutes that correlate with colorectal cancer (CRC) by inducing some oncogenic miRNAs such as miR-503, miR-182and mir-17 ~ 92 cluster [199]. In this regard, Zhou et al. reported that tumor suppressor miR-203, which targets CASK oncogene, can be downregulated by *Helicobacter pylori* infection and promoted the proliferation and invasion of gastric cancer [200]. Recent findings of the association between host miRNAs and parasites have shown that they can play reciprocal roles in this regard to modulate pathogenesis. For example, mmu-miR-101b-3p is increased in the infection of a nematode (larvae), *Angiostrongylus cantonensis*, and could reduce the pathological effect of the parasite in the host by targetting extracellular superoxide dismutase 3 (Acsod3*) *in vitro and in vivo [201]. Furthermore, miR-146a and miR-155 as a biomarker are upregulated in Toxoplasmosis that modulates inflammatory factors in hosts [202]. Also, *Toxoplasma gondii* infection can alter the expression levels of miR-17 ~ 92 and miR-106b ~ 25 clusters that contribute to enhancing the related diseases [203].

On the other hand, some miRNAs of parasites can also impact the host cells. For instance, the extracellular vesicle (EVs) miRNAs cargo like miR-125b and bantam miRNAs derived from *Schistosoma japonicum* leads to an increase in TNF-α production and macrophage proliferation in host cells by targeting and regulating Fam212b, Pros1, and Clmp; then, it elevates the rate of survival of the parasite in mouse [204]. Overall, various studies highlighted the issue that the ability of host cellular miRNA networks as a tool may control xeno-infection dissemination. The host’s miRNAs as immunomodulatory agents may target some pathogenic factors. On the other hand, because of the conserved properties of miRNAs in different organisms, some parasite-derived miRNAs may target the host’s transcripts. Therefore, there are reciprocally associations between host miRNAs and related infectious agents.

### Future perspective

Given the aforementioned contents on miRNA properties, the precise studies of isomiR profiling, arm selection, and arm switching can be applied to the related diseases that need reasonable and advantageous methods for their specific detection to be used as prognostic and diagnostic markers. Besides, the impressive variants of miRNAs are significant in miRNAs-targeted therapy. Ideal characteristics of miRNA and their related isomiRs, as well as their dependency on individual characteristics such as population origin, race, a person’s sex, and on tissue state/type, will provide an improvement in comprehension of the molecular mechanisms of diseases. Also, it provides new insights into novel approaches to improve personalized medicine. However, it still needs further investigation [205–207]. Furthermore, owing to epigenetically controlling miRNAs in their processing and expression levels, epigenetic-controlling agents can be used for regulation of them. With regard to the relationship between lncRNA and miRNA, lncRNAs can be used to control sponge-related miRNAs as a therapeutic strategy that may be considered another way for regulating miRNAs in various diseases. Finally, considering the roles of miRNA in xeno-infectious diseases, it can be controlled with mentioned approaches to attenuate infections.

## Conclusion

To highlight the role of miRNAs in the related diseases, some of them are collected in this review. Numerous studies have established that miRNAs are involved in both spectra of normal biological functions and diseases by directly or indirectly regulating multiple cellular transcripts by affecting epigenetic-related enzymes, thereby interfering with lncRNA functions with CeRNA roles. Also, they modulate xeno-infectious diseases by host and/or infection factors such as related transcripts and proteins, etc. On the other hand, complexities in the structure of their miRNAs such as isomiRs, arm selection, and arm switching can demonstrate the critical roles of miRNAs in the development of various diseases. Regarding the combination of structural properties of miRNAs and their interaction with epigenetics and other non-coding intracellular RNAs, they can also be affected by xeno-infectious agents such as viruses, parasites and bacteria, etc. Therefore, the potential role of miRNAs should be further considered because they are valuable prognostic and diagnostic biomarkers. In addition, to be applied as therapeutic agents, further studies are needed to be conducted from bench to bedside because miRNAs provide new insights into some mechanisms of complex diseases such as cancer, as well as neurodegenerative and xeno-infectious diseases that can be efficiently used in personalized medicine to control the diseases.

## Data Availability

Please contact the corresponding author for data requests.

## Abbreviations

Acsod3: Extracellular superoxide dismutase 3
ADR: RNA-specific adenosine deaminase
Ago: Argonaute protein
ARS: Arm switching
BoDV-1: Borna disease virus 1
BRD4: Bromo domain-containing protein 4
CASMIR: Comprehensive Approach to Sequence-oriented isomiR annotation
CCA: Cholangiocarcinoma
CLL: Chronic lymphocytic leukemia
CRC: Colorectal cancer
DENV: Dengue virus
DGCR8: DiGeorge syndrome critical region 8
DNMT: DNA methyltransferase
EMT: Epithelial-Mesenchymal Transition
ESCC: Esophageal squamous cell carcinoma
ES: Embryonic Stem
Evs: Extracellular vesicle
EV71: Enterovirus 71
FN1: Fibronectin 1
GBM: Glioblastoma
HBV: Hepatitis B virus
HBx: Hepatitis B virus X protein
HCV: Hepatitis C virus
HDAC4: Histone deacetylase 4
HP1α: Heterochromatin protein 1α
HSCs: Hepatic stellate cells
HSV-1: Herpes Simplex Virus-1
HTLV-1: Human T cell leukemia virus
HuR: Human antigen R, type 1
IAV: Influenza A virus
Lnc: Long Non-Coding
MBDs: Methyl-CpG-binding domains
MeCP2: Methyl-CpG-binding protein 2
miRNA: MicroRNA
MMP: Matrix metalloproteinase
m6A: N6-methyladenosine
m7G: N7-methylguanosine
NDV: Newcastle Disease Virus
OC: Ovarian cancer
pre-miRNA: Precursor miRNA
PACT: Protein activator of dsRNA-dependent protein kinase
RISC: RNA-induced silencing complex
SETDB1: SET domain bifurcated 1
SP1: Specificity protein 1
SRP14: Signal recognition particle 14
TCA: Tricarboxylic acid
TET: Ten-eleven translocation
TRBP: Trans-activating response RNA-binding protein
XPO5: Exportin-5
Xrn: 5′-3′ Exoribonuclease
YWHAZ: Tyrosine3 monooxygenase/tryptophan5-monooxygenase activation protein zeta
